# Case Report: Gastric and duodenal metastasis of malignant melanoma: a rare clinical presentation

**DOI:** 10.3389/fonc.2025.1597380

**Published:** 2025-10-09

**Authors:** Huantian Zheng, Weijian Zhang, Weiqin Yang, Lingyun Liu, Yu Peng, Yanzi Huang, Shaogang Huang, Jianyuan Kang, Baofu Lin, Shaoju Guo, Haiwen Li

**Affiliations:** ^1^ Department of Gastroenterology, Shenzhen Traditional Chinese Medicine Hospital, The Fourth Clinical Medical College of Guangzhou University of Chinese Medicine, Shenzhen, China; ^2^ The Eighth Affiliated Hospital, Sun Yat-Sen University, Chinese Medicine Department, Shenzhen, China; ^3^ Department of Pathology, Shenzhen Traditional Chinese Medicine Hospital, The Fourth Clinical Medical College of Guangzhou University of Chinese Medicine, Shenzhen, China; ^4^ Science and Technology Innovation Center, Guangzhou University of Chinese Medicine, Guangzhou, China; ^5^ The First Affiliated Hospital of Guangzhou University of Chinese Medicine, Guangzhou University of Chinese Medicine, Guangzhou, China

**Keywords:** malignant melanoma, gastric and duodenal metastasis, endoscopic evaluation, immunohistochemical analysis, prognosis

## Abstract

**Background:**

Malignant melanoma represents one of the most common sources of metastatic tumors to the gastrointestinal (GI) tract. However, synchronous involvement of both the stomach and duodenum is exceptionally rare. Ante-mortem diagnosis remains challenging due to frequent asymptomatic or non-specific presentations. Endoscopically, metastases may present as ulcerated nodules, submucosal masses, or pigmented lesions, necessitating confirmation via immunohistochemical staining.

**Objective:**

This case report describes a rare instance of synchronous gastric and duodenal metastases from malignant melanoma, aiming to enhance clinical awareness of this condition.

**Methods:**

We present the case of a 67-year-old male with a history of wild-type BRAF V600E malignant melanoma of the left lower limb, status post resection three years prior, who presented for observation with known multi-system metastases. The patient reported decreased appetite but denied other GI symptoms. Upper gastrointestinal endoscopy was performed, revealing suspicious lesions in the stomach and duodenum, which were subsequently biopsied for histopathological and immunohistochemical analysis.

**Results:**

Endoscopy identified a mass on the posterior wall of the gastric fundus and the greater curvature of the upper stomach, alongside four masses in the duodenal bulb. All lesions exhibited surface melanin deposition. Histological examination revealed tumor cells with prominent nucleoli and visible melanin granules. Immunohistochemistry was positive for S100, Melan-A, and SOX10, with a high Ki67 proliferation index of 90%, confirming the diagnosis of metastatic malignant melanoma.

**Conclusion:**

This case underscores the potential for malignant melanoma to develop synchronous metastases in both the stomach and duodenum, even in the absence of specific GI symptoms (3, 6). It highlights the critical role of endoscopic evaluation and immunohistochemical analysis in achieving a timely diagnosis (4, 7). A high index of suspicion is warranted in patients with a history of melanoma, as GI metastases confer a poor prognosis (4, 7).

## Objectives

This case report aims to: (1) document the clinicopathological features of exceptionally rare synchronous gastric and duodenal metastases from malignant melanoma; (2) emphasize the importance of including metastatic melanoma in the differential diagnosis for patients with relevant history, regardless of gastrointestinal symptoms; and (3) highlight the indispensable role of endoscopic surveillance coupled with immunohistochemical profiling for accurate diagnosis.

## Case report

**Figure 1:**
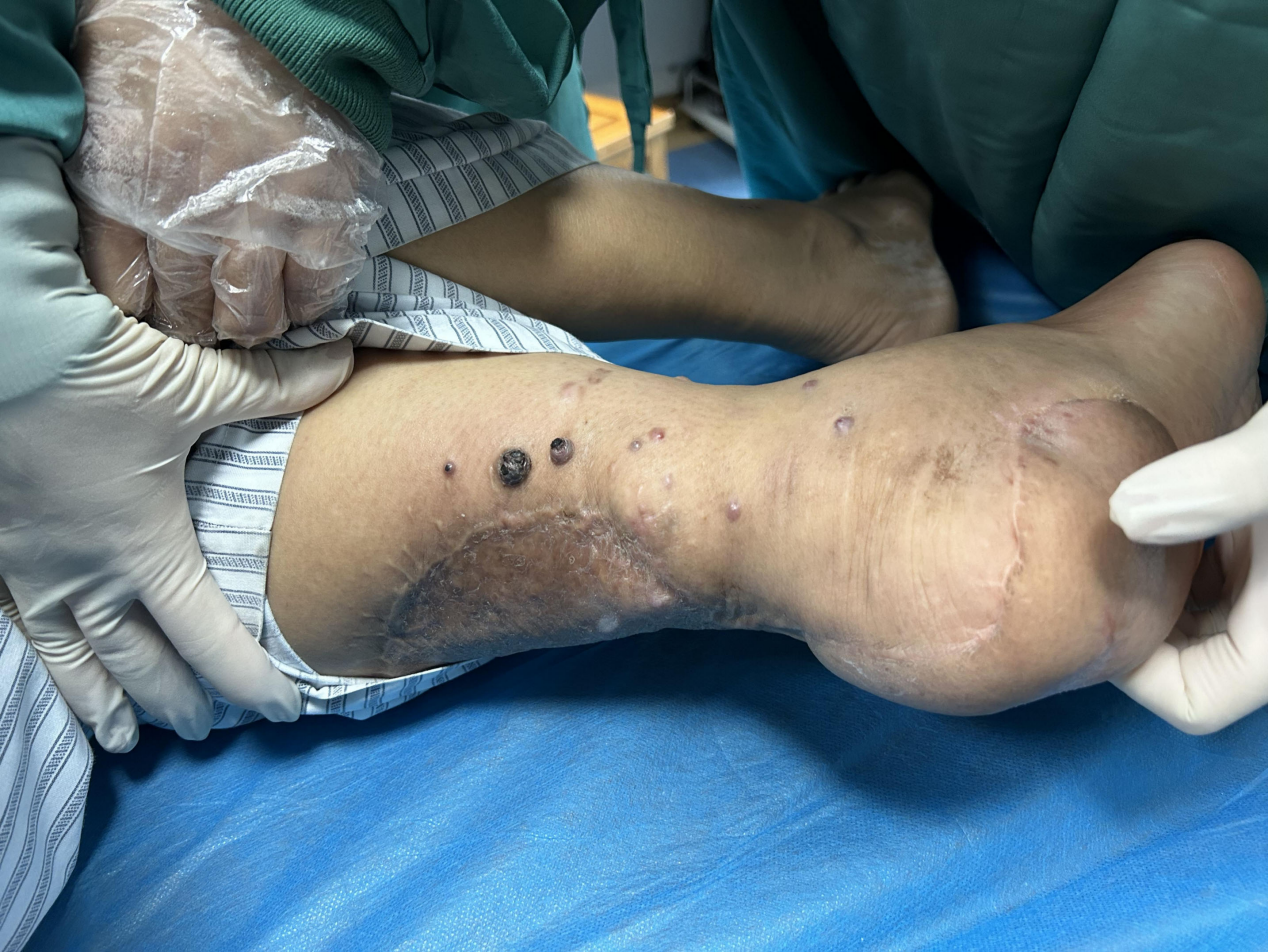
Physical examination shows multiple melanoma in the left lower limb.

**Figure 2:**
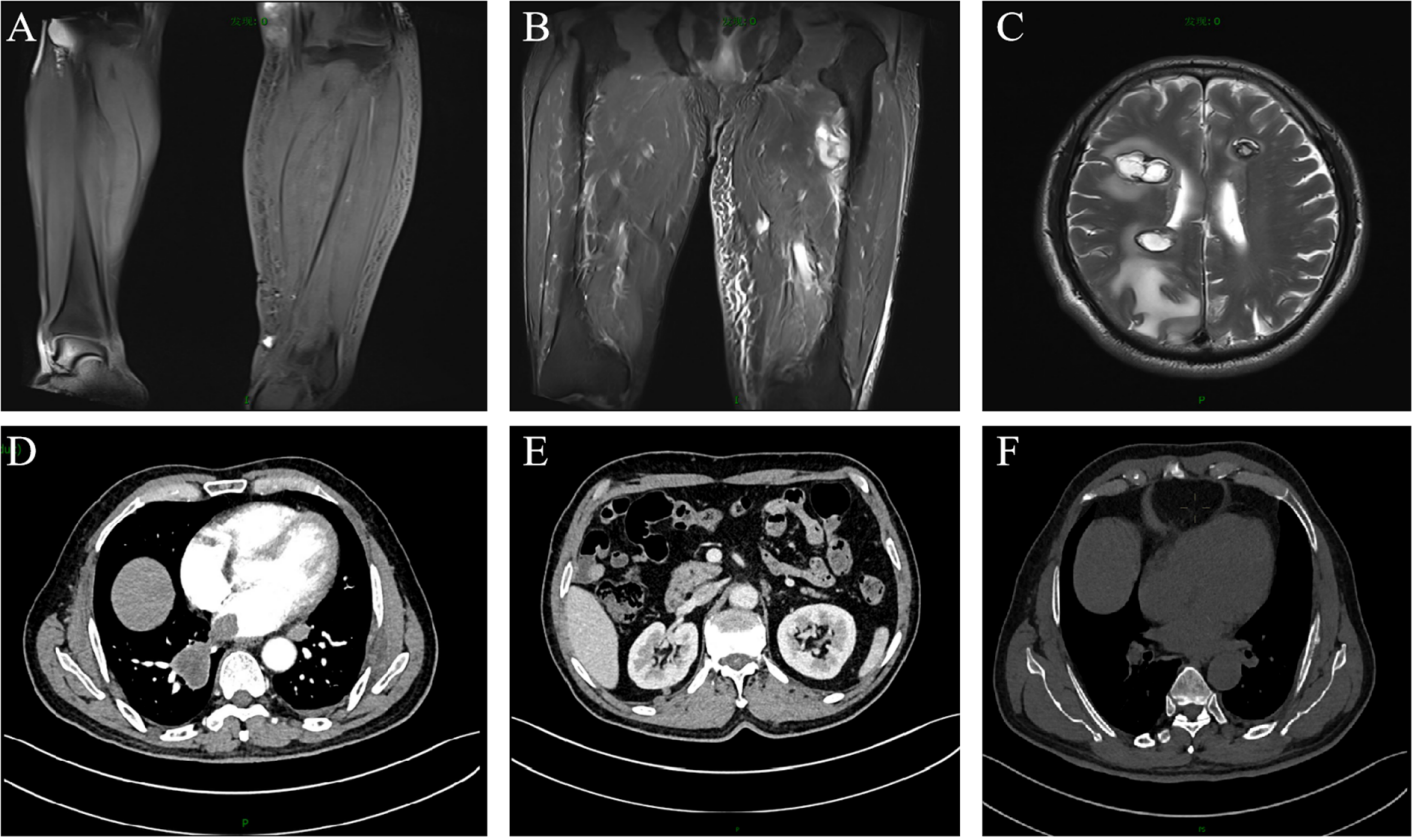
Enhanced magnetic resonance imaging and computed tomography scans of the patient. Metastatic lesions in the: **(A)** left calf; **(B)** left thigh; **(C)** brain; **(D)** lungs and pericardium; **(E)** kidneys; **(F)** bones.

**Figure 3:**
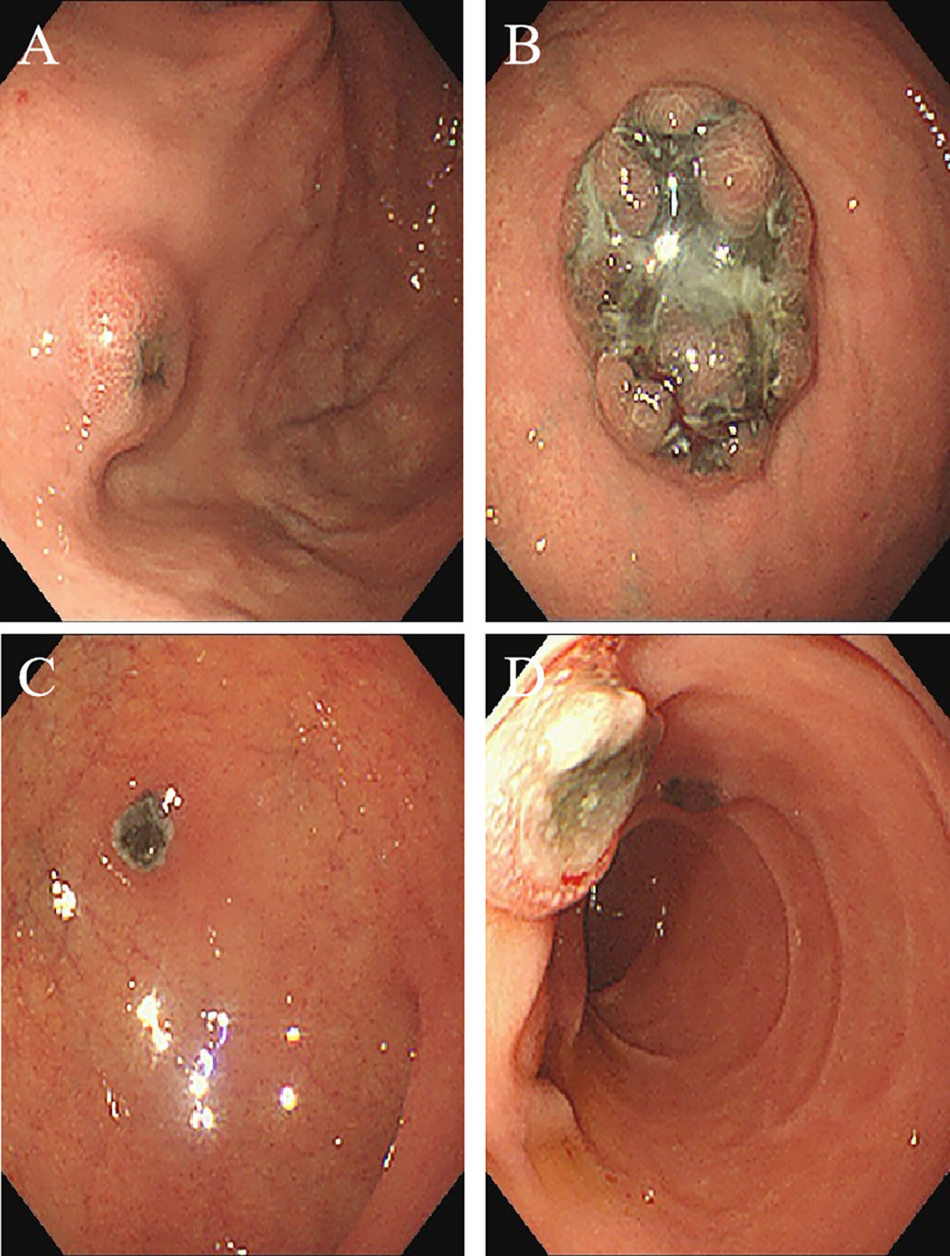
Endoscopic imaging shows multiple metastatic melanomas in the: **(A)** posterior wall of gastric fundusstomach; **(B)** greater curvature of the upper part of the stomach; **(C)** anterior wall of duodenal bulb; **(D)** descending segment of duodenum.

**Figure 4:**
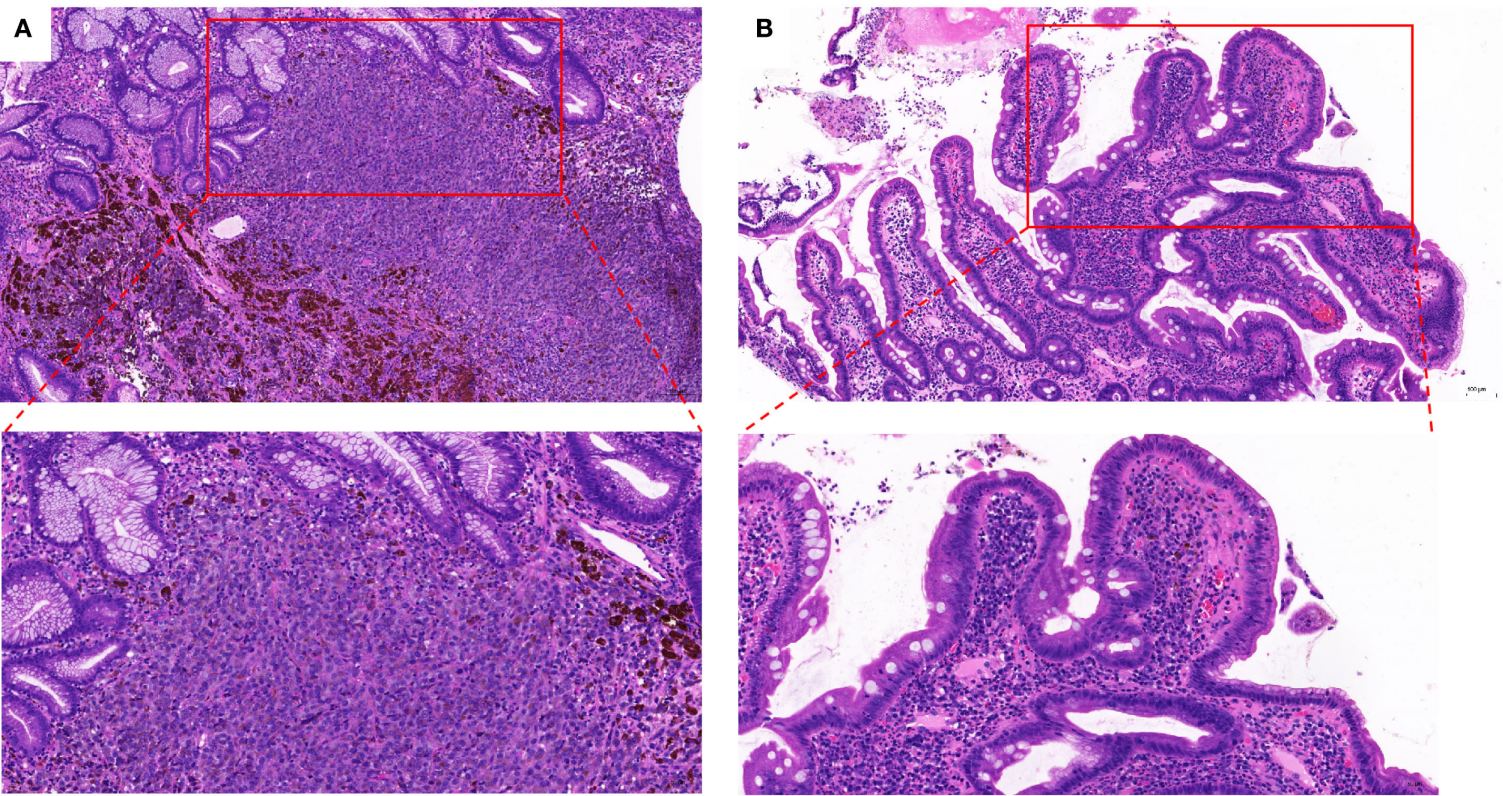
Stained with hematoxylin and eosin showing for **(A)** gastric submucosa (X 100 and X 200); **(B)** Duodenal mucosa (X 100 and X 200).

**Figure 5:**
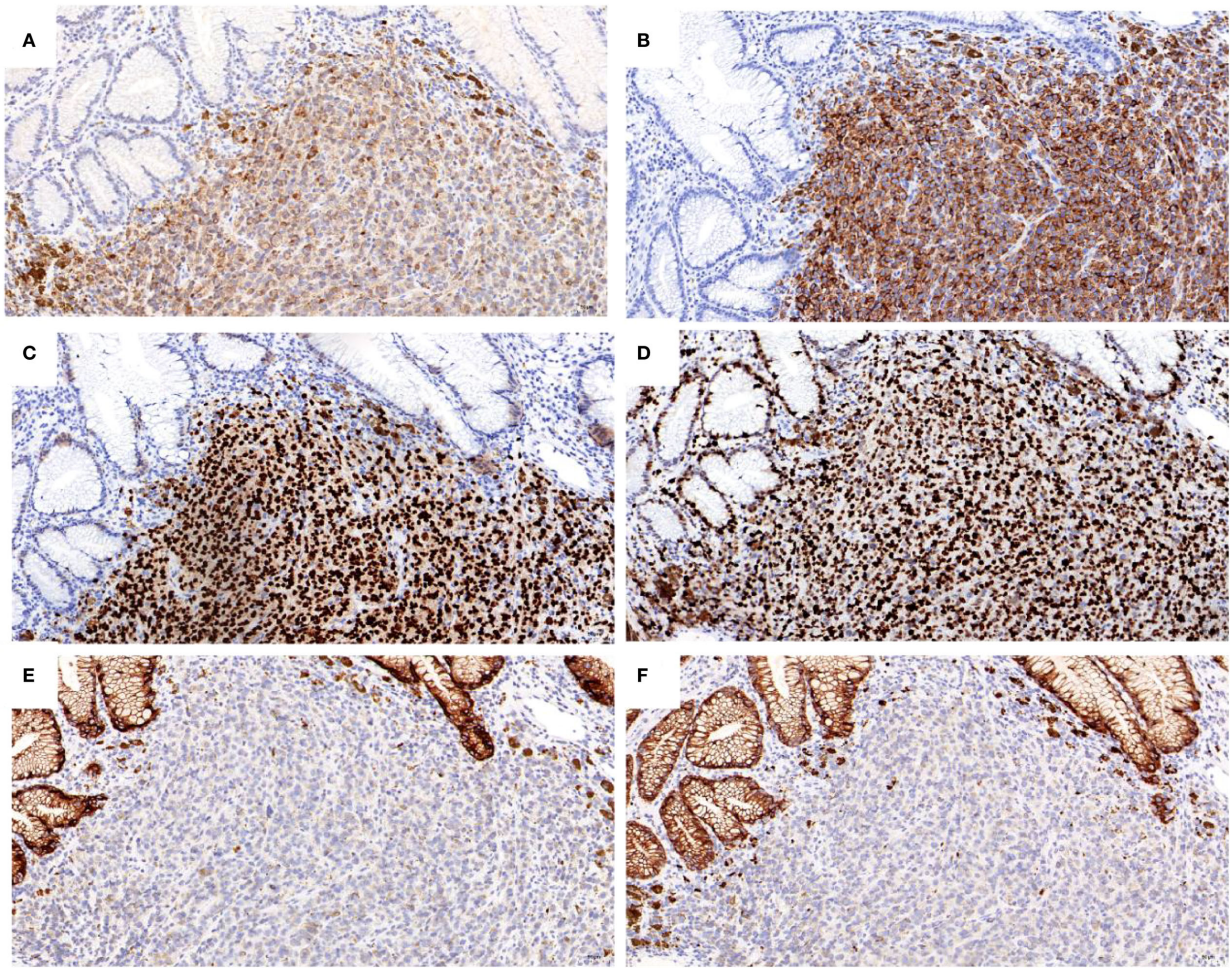
Immunohistochemical analysis of the tumor cells for positive staining of: **(A)** S100; **(B)** Melan A; **(C)** SOX10; **(D)** KI67 expression is about 90%; negative staining of: **(E)** CK; **(F)** CAM5.2 (X 200).

A 67-year-old male patient presented with melanoma of the left lower limb (**Figure 1**), accompanied by multiple systemic metastases, including those in the brain, lungs, pericardium, kidneys, and bones, and sought medical observation at our hospital (**Figure 2**). Three years prior, he underwent surgical resection and was subsequently diagnosed with wild-type BRAF V600E through pathological examination. He is currently undergoing regular immunotherapy with trastuzumab. Over the past year, the patient has reported a decrease in appetite but has not experienced any abdominal symptoms such as pain, nausea, vomiting, bloody stools, weight loss, or anemia. During an upper gastrointestinal endoscopy, a mass was identified on the posterior wall of the gastric fundus and the greater curvature of the upper stomach, with melanin deposition observed on the surface (**Figures 3A, B**). Additionally, four masses were detected on the anterior wall and descending segment of the duodenal bulb, also exhibiting surface melanin deposition (**Figures 3C, D**). Consequently, a histological examination of the masses located in both the stomach and the duodenal bulb was deemed necessary. Histological examination using hematoxylin and eosin staining revealed numerous tumor cells of varying sizes with prominent nucleoli in the local gastric mucosal tissue, alongside some cells containing visible melanin granules (**Figure 4A**). High magnification analysis of the duodenum also identified a small quantity of pigments in the localized area, which are characteristic features of melanoma (**Figure 4B**). Immunohistochemical analysis demonstrated positive expression for S100, MelanA, and SOX10 (**Figures 5A–C**), while CK and CAM5.2 the result turns out negative (**Figures 5D–F**). The Ki67 proliferation index was 90%, aligning with the diagnosis of metastatic malignant melanoma. The patient declined additional treatment and was subsequently discharged.

## Discussion

Malignant melanoma constitutes one of the most frequent origins of gastrointestinal (GI) tract metastases (1). Nevertheless, synchronous involvement of both the stomach and duodenum is highly uncommon (2). Ante-mortem diagnosis is often challenging, as patients may remain asymptomatic or present with non-specific symptoms (3). Endoscopic findings can include ulcerated nodules, submucosal masses, or pigmented lesions, with definitive diagnosis relying on immunohistochemical analysis (4). Histological assessment typically demonstrates tumor cells featuring prominent nucleoli and melanin granules, while immunohistochemical staining is positive for S100, Melan-A, and SOX10. A high Ki67 proliferation index (e.g., 90%) further supports the diagnosis of metastatic malignant melanoma (4, 5). This case highlights the potential for malignant melanoma to produce synchronous gastric and duodenal metastases, even in the absence of specific GI manifestations (3, 6). It also underscores the essential role of endoscopic examination and immunohistochemical profiling in establishing a timely diagnosis (4, 7). A high clinical suspicion is warranted in patients with a history of melanoma, given that GI metastases are associated with a poor prognosis (4, 7). This case report details a rare instance of malignant melanoma with concurrent gastric and duodenal metastases. Malignant melanoma is a highly aggressive malignant tumor characterized by its potential for multiorgan metastasis, with the gastrointestinal (GI) tract being one of the commonly involved sites (8). According to literature, melanoma most frequently metastasizes to the small intestine, followed by the stomach and colon (9). The duodenum, as a key anatomical segment of the small intestine, is also frequently involved (2). It is noteworthy that isolated gastric metastases are clinically rare, and even among patients with advanced melanoma, the detection rate of gastric involvement remains very low (3, 4, 10). This low incidence is largely attributable to the frequent absence of symptoms or non-specific clinical manifestations, which often leads to underdiagnosis (3). Furthermore, synchronous gastric and duodenal metastases are exceptionally uncommon in clinical practice, posing additional diagnostic and therapeutic challenges (2).The clinical manifestations of gastric and duodenal metastases from malignant melanoma are non-specific. Common symptoms include abdominal pain, gastrointestinal bleeding, nausea and vomiting, early satiety, weight loss, and anemia-related symptoms (6, 11). These manifestations can easily be mistaken for other gastrointestinal disorders, leading to delays in diagnosis (3). In terms of imaging modalities, PET/CT offers significant advantages in detecting systemic metabolically active lesions, making it highly valuable for metastasis screening (7, 12). In contrast, contrast-enhanced CT (CECT) is more widely used in clinical settings due to its greater availability, faster scanning time, lower cost, and reduced radiation exposure (13, 14). Regarding endoscopic strategies, colonoscopy is not recommended as a first-line screening tool for asymptomatic melanoma patients without clinical manifestations or imaging evidence of colonic involvement (15). However, it is important to note that melanoma metastases are often multifocal (5). If imaging reveals abnormalities in the colon—such as suspicious wall thickening in the sigmoid colon, as observed in this case—colonoscopy should be recommended to allow comprehensive evaluation of the entire gastrointestinal tract (16, 17). In this patient, colonoscopy was ultimately declined due to concerns regarding their physical condition and the invasive nature of the procedure, highlighting the need to balance standardized diagnostic protocols with individual patient preferences. Follow-up intervals for patients with malignant melanoma should be individualized based on disease burden, treatment regimen, and clinical status (18). We recommend that during active treatment, imaging is generally performed every 2–4 months to closely monitor disease progression (18). Once stability is achieved, the interval may be gradually extended to every 3–6 months (3, 7, 19). Any new or worsening symptoms should prompt immediate imaging investigation to assess potential disease progression (20). In terms of treatment, the management of metastatic melanoma has undergone a paradigm shift. Surgery is no longer considered the primary or “best” treatment option for most patients with disseminated metastatic disease (8). Current standard care relies on systemic therapy, mainly comprising immunotherapy and targeted therapy (8). It is particularly important to emphasize that radiotherapy (RT) is not a first-line treatment for GI metastases of melanoma, primarily because these lesions are often multifocal and disseminated, and the required radiation dose can cause significant damage to adjacent normal intestinal tissues (21). Regarding diagnostic differentiation, it is crucial to distinguish between primary gastric melanoma and metastatic gastric melanoma (22). Primary gastric melanoma is exceedingly rare and is essentially a diagnosis of exclusion (4, 7). Its confirmation must meet the following three criteria: first, the presence of a solitary gastric lesion exhibiting typical histological features of melanoma; second, the comprehensive exclusion of other primary sites through a full dermatological examination, ophthalmologic evaluation, and review of any prior lesions (7); and third, the demonstration of an *in situ* melanoma component in the overlying or adjacent gastric mucosa-a feature that is often absent (7). In contrast, the vast majority of melanomas found in the stomach are metastatic, originating from cutaneous or mucosal sites (such as the sinonasal region or anorectal area) or arising from an unknown primary (2, 8). In the present case, the diagnosis of metastatic disease was unequivocal, given the patient’s history of primary cutaneous melanoma and the presence of multifocal lesions, thereby ruling out the possibility of primary gastric melanoma. Literature indicates that melanoma metastasis to the gastrointestinal tract is associated with a median survival period of 4–6 months and a notably poor prognosis (11, 22, 23). The patient in this case exhibited rapid disease progression and succumbed to the illness within seven months after diagnosis, which is consistent with the data reported in the literature and highlights the significant therapeutic challenges posed by this type of metastasis. It is recommended that patients with malignant melanoma undergo endoscopic screening to rule out gastrointestinal metastasis, irrespective of the presence of specific abdominal symptoms. Early detection and surgical intervention significantly enhance the prognosis of individuals diagnosed with melanoma. (Informed consent was obtained from the patient to publish these images).

## Data Availability

The datasets presented in this study can be found in online repositories. The names of the repository/repositories and accession number(s) can be found in the article/supplementary material.
